# Supplementary material for: Triphenilphosphonium Analogs of Chloramphenicol as Dual-Acting Antimicrobial and Antiproliferating Agents
Source: Antibiotics (Basel). 2021 Apr 23;10(5):489. doi: 10.3390/antibiotics10050489 (PMC8145938; doi:10.3390/antibiotics10050489)
Supplement: Supplementary file 1 [file antibiotics-10-00489-s001.zip › antibiotics-1186843-supplementary.pdf]

antibiotics

MDPI

Article

Triphenilphosphonium Analogs of Chloramphenicol as Dual-Acting Antimicrobial and Antiproliferating Agents

Julia A. Pavlova, Zimfira Z. Khairullina, Andrey G. Tereshchenkov, Pavel A. Nazarov, Dmitrii A. Lukianov, Inna A. Volynkina, Dmitry A. Skvortsov, Gennady I. Makarov, Etna Abad, Somay Y. Murayama, Susumu Kajiwara, Alena Paleskava, Andrey L. Konevega, Yuri N. Antonenko, Alex Lyakhovich, Ilya A. Osterman, Alexey A. Bogdanov and Natalia V. Sumbatyan

Supplementary Methods

Detailed synthesis of CAM-C14-TPP

4-(tert-Butoxycarbonylamino)-N-[(1R,2R)-1,3-dihydroxy-1-(4-nitrophenyl)propan-2-yl]butanamide (Boc-GABA-CAM). 792 mg (3.84 mmol) of *N,N'*-dicyclohexylcarbodiimide (DCC) was added to a cold solution of 600 mg (2.96 mmol) of 4-(*tert*-butoxycarbonylamino)butyric acid (Boc-GABA) and 442 mg (3.84 mmol) of *N*-hydroxysuccinimide (HOSu) in 15 ml of anhydrous DMF at 0 °C. The mixture was stirred for 2 h at 0 °C, then overnight at 4 °C. After that 736 mg (2.96 mmol) of chloramphenicol amine hydrochloride (CAM, **2**) and 516 µl (2.96 mmol) of DIPEA was added and the resulting mixture was stirred overnight at RT. The formed precipitate was filtered off, the reaction mixture was diluted with 200 ml of water, and the product was extracted with ethyl acetate (3 × 50 ml). Organic layer was washed with 0.05 M solution of H_2SO_4 (3 × 50 ml), water (1 × 50 ml), 5% solution of $NaHCO_3$ (3 × 50 ml), and saturated NaCl (1 × 30 mL). Then ethyl acetate was dried over anhydrous Na_2SO_4 and evaporated in vacuo. The target product was isolated from the residue by purification on silica gel column eluting with solvents system CH_2Cl_2 : MeOH, 9:1. Yield: 946 mg (80%); TLC: R_f ($CHCl_3$: MeOH, 9:1) 0.34; LC-MS m/z calculated for $C_{18}H_{28}N_3O_7$ $(M+H)^+$ 398.19, found 398.41; t_R = 1.57 min. ^1H NMR (DMSO-d_6, 400 MHz) δ (ppm) 8.18–8.11 (m, 2H, NO_2-Ph_{ortho}), 7.60–7.55 (m, 2H, NO_2-Ph_{meta}), 7.53 (d, J = 9.2 Hz, 1H, -CH-N<u>H</u>-C(O)-), 6.70 (t, J = 5.7 Hz, 1H, Boc-N<u>H</u>-), 5.83 (d, J = 4.7 Hz, 1H, -CH-O<u>H</u>), 5.01 (dd, J = 5.5, 4.7 Hz, 1H, -C<u>H</u>-OH), 4.85 (dd, J = 6.4, 4.3 Hz, 1H, -CH_2-O<u>H</u>), 4.01–3.94 (m, 1H, -C<u>H</u>-NH-C(O)-), 3.54 (ddd, J = 10.4, 8.2, 6.4 Hz, 1H, -C<u>H</u>$_2^a$-OH), 3.33–3.25 (m, 1H, -C<u>H</u>$_2^b$-OH), 2.69 (q, J = 6.8 Hz, 2H, Boc-NH-C<u>H</u>$_2$-), 1.97 (t, J = 7.4 Hz, 2H, -CH_2-C<u>H</u>$_2$-C(O)-), 1.44–1.38 (m, 2H, -CH_2-C<u>H</u>$_2$-CH_2-), 1.36 (s, 9H, -C<u>H</u>$_3$). ^{13}C NMR (DMSO-d_6, 101 MHz) δ (ppm) 171.80 (-CH_2-<u>C</u>(O)-), 157.69 (-O-<u>C</u>(O)-NH-), 152.06 (NO_2-Ph_{ipso}), 146.32 (NO_2-Ph_{para}), 127.34 (2C, NO_2-Ph_{meta}), 122.82 (2C, NO_2-Ph_{ortho}), 77.44 (-<u>C</u>(CH_3)$_3$), 69.33 (-<u>C</u>H-OH), 60.58 (-<u>C</u>H_2-OH), 55.84 (-NH-<u>C</u>H-), 39.31 (-NH-<u>C</u>H_2-), 32.66 (-<u>C</u>H_2-C(O)-), 28.29 (3C, -<u>C</u>H_3), 25.89 (-CH_2-<u>C</u>H_2-CH_2-).

4. -amino-N-[(1R,2R)-1,3-dihydroxy-1-(4-nitrophenyl)propan-2-yl]butanamide (GABA-CAM, 6).

10 ml of 50% (*v*/*v*) TFA solution in CH_2Cl_2 was added to 846 mg of Boc-GABA-CAM. The mixture was stirred for 30 min at room temperature and then evaporated in vacuo. The target product was isolated by purification on silica gel column eluting with solvents system CH_2Cl_2: MeOH : NH_4OH, 65:25:4. Yield: 620 mg (98%); TLC: R_f ($CHCl_3$: MeOH : NH_4OH, 65:25:4) 0.20; LC-MS m/z calculated for $C_{13}H_{20}N_3O_5$ $(M+H)^+$ 298.14, found 298.39; t_R = 0.49 min. ^1H NMR (DMSO-d_6, 400 MHz) δ (ppm) 8.15 (d, J = 8.8 Hz, 2H, NO_2-Ph_{ortho}), 7.68 (d, J = 9.2 Hz, 1H, -N<u>H</u>-C(O)-), 7.58 (d, J = 8.8 Hz, 2H, NO_2-Ph_{meta}), 5.92 (d, J = 5.4 Hz, 1H, -CH-O<u>H</u>), 5.02 (s, 1H, -C<u>H</u>-OH), 4.97 (br. s, 1H, -CH_2-O<u>H</u>), 4.05–3.94 (m, 1H, -C<u>H</u>-NH-C(O)-), 3.56 (dd, J = 10.5, 7.9 Hz, 1H, -C<u>H</u>$_2^a$-OH), 3.31 (dd, J = 10.5, 6.0 Hz, 1H, -C<u>H</u>$_2^b$-OH),

2.65–2.55 (m, 2H, NH$_2$-C$\underline{H}_2$-), 2.10 (t, J = 7.2 Hz, 2H, -CH$_2$-C$\underline{H}_2$-C(O)-), 1.60 (p, J = 7.6 Hz, 2H, -CH$_2$-C$\underline{H}_2$-CH$_2$-). ^{13}C NMR (DMSO-d_6, 101 MHz) δ (ppm) 171.45 (-CH$_2$-$\underline{C}$(O)-), 152.13 (NO$_2$-Ph$_{ipso}$), 146.51 (NO$_2$-Ph$_{para}$), 127.51 (2C, NO$_2$-Ph$_{meta}$), 122.96 (2C, NO$_2$-Ph$_{ortho}$), 69.63 (-$\underline{C}$H-OH), 60.77 (-$\underline{C}$H$_2$-OH), 56.13 (-NH-$\underline{C}$H-), 35.50 (NH$_2$-$\underline{C}$H$_2$-), 32.15 (-$\underline{C}$H$_2$-C(O)-), 23.47 (-CH$_2$-$\underline{C}$H$_2$-CH$_2$-).

N-[(1R,2R)-1,3-dihydroxy-1-(4-nitrophenyl)propan-2-yl]-4-(triphenyl)phosphoniumundecanamidobutamide bromide (CAM-C14-TPP)

To the cold solution of 263 mg (0.5 mmol) of (10-carboxydecyl)(triphenyl)phosphonium bromide **(5)** and 58 mg (0.5 mmol) of *N*-hydroxysuccinimide in 7 ml of anhydrous CH_2Cl_2 103 mg (0.5 mmol) of DCC was added at 0 °C. The mixture was stirred for 2 h at 0 °C and overnight at RT. Then 149 mg (0.5 mmol) of GABA-CAM (**6**) was added to the mixture and stirred for 5h at RT and overnight at 4 °C. The reaction mixture was diluted with 15 ml of water and 1N aqueous HCl was added dropwise to neutral pH. Then the product was extracted with $CHCl_3$ (3 × 15 ml), and the combined organic extracts were washed with water (3 × 10 ml). Organic layer was dried over anhydrous Na_2SO_4, the volatiles were evaporated in vacuo. The target product was isolated on silica gel column eluting with solvents system $CHCl_3$: MeOH : NH_4OH = 65:25:4. Yield: 261 mg (65%); TLC: R_f ($CHCl_3$: MeOH : NH_4OH, 65:25:4) 0.7; LC-MS m/z calculated for $C_{42}H_{53}N_3O_6P$ (M)$^+$ 726.4, found 726.7; t_R = 0.50 min; ESI-MS m/z calculated for $C_{42}H_{53}N_3O_6P$ (M)$^+$: 726.3667, found 726.3692. ^{1}H NMR (DMSO-d_6, 400 MHz) δ (ppm) 8.12 (d, J = 8.8 Hz, 2H, NO$_2$-Ph$_{ortho}$), 7.89 (td, J = 7.0, 1.9 Hz, 3H, -Ph$_{para}$), 7.85–7.73 (m, 12H, -Ph$_{ortho}$, -Ph$_{meta}$), 7.70 (t, J = 5.4 Hz, 1H, -CH$_2$-N$\underline{H}$-), 7.65 (d, J = 9.0 Hz, 1H, -CH-N$\underline{H}$-), 7.58 (d, J = 8.8 Hz, 2H, NO$_2$-Ph$_{meta}$), 6.06 (br. s, 1H, -CH-O$\underline{H}$), 5.03 (d, J = 2.5 Hz, 1H, -C$\underline{H}$-OH), 4.97–4.84 (m, 1H, -CH$_2$-O$\underline{H}$), 4.04–3.90 (m, 1H, -C$\underline{H}$-NH-), 3.61–3.48 (m, 3H, Ph$_3$P$^+$-C$\underline{H}_2$-, -C$\underline{H}_2^a$-OH), 3.28 (dd, J = 10.3, 5.8 Hz, 1H, -C$\underline{H}_2^b$-OH), 2.79 (td, J = 12.3, 6.4 Hz, 2H, -NH-C$\underline{H}_2$-), 2.06–1.89 (m, 4H, -NH-C(O)-C$\underline{H}_2$-), 1.76–1.56 (m, 2H, -C(O)-CH$_2$-C$\underline{H}_2$-CH$_2$-NH-), 1.56–1.06 (m, 16H, -C$\underline{H}_2$-). ^{13}C NMR (DMSO-d_6, 101 MHz) δ (ppm) 171.95 (-CH-NH-$\underline{C}$(O)-), 171.79 (-CH$_2$-NH-$\underline{C}$(O)-), 152.21 (NO$_2$-Ph$_{ipso}$), 146.23 (NO$_2$-Ph$_{para}$), 134.91 (d, $J_{C,P}$ = 2.9 Hz, 3C, -Ph$_{para}$), 133.61 (d, $J_{C,P}$ = 10.1 Hz, 6C, -Ph$_{ortho}$), 130.27 (d, $J_{C,P}$ = 12.4 Hz, 6C, -Ph$_{meta}$), 127.37 (2C, NO$_2$-Ph$_{meta}$), 122.76 (2C, NO$_2$-Ph$_{ortho}$), 118.62 (d, $J_{C,P}$ = 85.7 Hz, 3C, -Ph$_{ipso}$), 69.35 (-$\underline{C}$H-OH), 60.55 (-C$\underline{H}_2$-OH), 55.91 (-$\underline{C}$H-NH-), 39.31 (-NH-$\underline{C}$H$_2$-), 37.86 and 35.42 (2C, 10-CH$_2$ and -NH-CH$_2$-CH$_2$-$\underline{C}$H$_2$-), 29.83 (d, $J_{C,P}$ = 16.6 Hz, 1C, 3-CH$_2$), 28.75, 28.68, and 28.09 (5C, 4, 5, 6, 7, and 8-$\underline{C}$H$_2$), 25.66 and 25.29 (2C, -NH-CH$_2$-$\underline{C}$H$_2$-CH$_2$ and 9-$\underline{C}$H$_2$), 21.74 (d, $J_{C,P}$ = 4.0 Hz, 1C, 2-CH$_2$), 20.14 (d, $J_{C,P}$ = 49.8 Hz, 1C, 1-CH$_2$). ^{31}P NMR (DMSO-d_6, 162 MHz) δ (ppm) 24.00.

NMR-data for CAM-C10-TPP

(N-{[(1R,2R)-1,3-dihydroxy-1-(4-nitrophenyl)propan-2-yl]amino}-11-oxoundecyl)(triphenyl)phosphonium bromide (CAM-C10-TPP).

^{1}H NMR (CDCl$_3$, 400 MHz) δ (ppm) 8.09 (d, J = 8.6 Hz, 2H, NO$_2$-Ph$_{ortho}$), 7.86–7.70 (m, 15H, -Ph), 7.63 (d, J = 8.6 Hz, 2H, NO$_2$-Ph$_{meta}$), 7.36 (d, J = 8.4 Hz, 1H, -N$\underline{H}$-C(O)-), 5.70 (br. s, 1H, -CH-O$\underline{H}$), 5.26 (d, J = 3.7 Hz, 1H, -C$\underline{H}$-OH), 4.62 (br. s, 1H, -CH$_2$-O$\underline{H}$), 4.22 (dddd, J = 8.4, 5.8, 4.7, 3.7 Hz, 1H, -C$\underline{H}$-NH-), 3.82 (dd, J = 11.7, 5.8 Hz, 1H, -C$\underline{H}_2^a$-OH), 3.66 (dd, J = 11.7, 4.7 Hz, 1H, -C$\underline{H}_2^b$-OH), 3.47 (dt, J = 12.6, 7.9 Hz, 2H, 1-C$\underline{H}_2$), 2.17 (t, J = 7.2 Hz, 2H, 10-C$\underline{H}_2$), 1.72–1.61 (m, 2H, 2-C$\underline{H}_2$), 1.58 (p, J = 7.4 Hz, 2H, 9-C$\underline{H}_2$), 1.47 (dp, J = 14.8, 7.4 Hz, 2H, 3-C$\underline{H}_2$), 1.36–1.03 (m, 10H, 4-, 5-, 6-, 7-, 8-C$\underline{H}_2$). ^{13}C NMR (CDCl$_3$, 151 MHz) δ (ppm) 174.24 (C=O), 151.10 (NO$_2$-Ph$_{ipso}$), 146.58 (NO$_2$-Ph$_{para}$), 135.33 (3C, d, $J_{C,P}$ = 3.1 Hz, Ph$_{para}$), 133.44 (6C, d, $J_{C,P}$ = 9.9 Hz, Ph$_{ortho}$), 130.64 (6C, d, $J_{C,P}$ = 12.6 Hz, Ph$_{meta}$), 127.38 (2C, NO$_2$-Ph$_{meta}$), 122.95 (2C, NO$_2$-Ph$_{ortho}$), 118.05 (3C, d, $J_{C,P}$ = 86.0 Hz, Ph$_{ipso}$), 71.63 (-$\underline{C}$H-OH), 62.03 (-$\underline{C}$H$_2$-OH), 56.33 (-NH-$\underline{C}$H-), 36.24 (10-$\underline{C}$H$_2$), 30.27 (d, $J_{C,P}$ = 15.8 Hz, 3-$\underline{C}$H$_2$), 28.84, 28.83, 28.77, 28.50, and 28.49 (5C, 4, 5, 6, 7, and 8-$\underline{C}$H$_2$), 25.49 (9-$\underline{C}$H$_2$), 22.52 (d, $J_{C,P}$ = 50.7 Hz, 1-$\underline{C}$H$_2$), 22.46 (d, $J_{C,P}$ = 4.5 Hz, 2-$\underline{C}$H$_2$). ^{31}P NMR (CDCl$_3$, 243 MHz) δ (ppm) 23.86.

Table S1. Occurrences of hydrogen bonds and stacking interactions of CAM-Cn-TPP, obtained by MD simulations (percent of frames). Chloramphenicol amine residue is designated as CAM, alkylated triphenylphosphonium fragment is designated as TPP and γ–aminobutyric acid residue of CAM-C14-TPP is designated as GABA.

Donor	Acceptor	CAM-C10-TPP	CAM-C14-TPP
	Hydrogen bonds		
CAM/O_3–H	G2505/$O_{2'}$	–	2
CAM/O_3–H	U2506/O^4	–	18
CAM/O_1–H	G2061/O^6	–	85
CAM/O_1–H	Ψ2504/$O_{2'}$	93	2
GABA/N–H	A2059/N^1	–	23
G2061/N^1–H	CAM/O_3	61	–
CAM/O_3–H	G2505/$O_{2'}$	–	2
	Stacking interactions		
CAM	Ψ2504	80	20
CAM	U2506	15	27

[1] We modeled the structures of CAM-C10-TPP and CAM-C14-TPP complexes with the *E. coli* ribosome in the canonical A,A/P,P–state, by sequentially docking these compounds into the upper part of the NPET and PTC fragments of A,A/P,P–ribosome, followed by equilibrium molecular dynamics simulations and clustering the obtained conformations by the GROMOS method. Analysis of the most populated clusters combining 55% of all obtained frames for the CAM-C10-TPP complex and 75% of all obtained frames for the CAM-C14-TPP complex revealed that these compounds should be able to interact stably with the non-canonical binding site of the CHL described in [59].

Table S2. Suppression of the growth of CHL resistant *E. coli* strains harbouring CHL acetyltransferase (*cat*) gene by CAM-C10-TPP (MIC, μM) [1].

	Escherichia coli res			
	J53rif	***C600rif/pIB55-1***	***C600rif/pIP162-1***	***C600recA naI***
CHL	>396	>396	>396	>396
CAM-10-TPP	110	90	>199	107
C10-TPP	110	67	93	136

[1] MIC values were determined using the double-dilution method. The MIC for each compound was determined in triplicate in two independent sets.

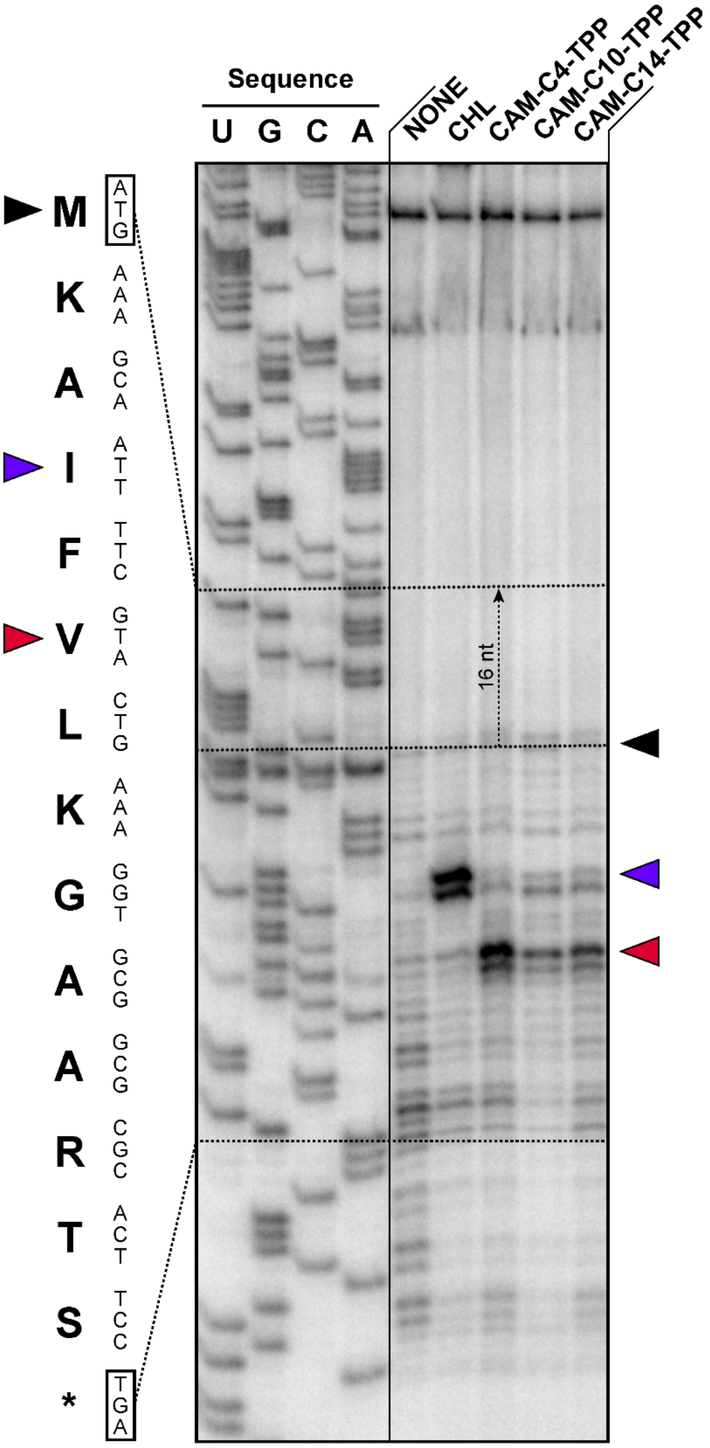

Figure S1. Original version of the Figure 2D. Ribosome stalling by CAM-Cn-TPP on *trpL* mRNA in comparison with CHL, revealed by reverse-transcription primer-extension inhibition (toeprinting) assay in a cell-free translation system. Nucleotide sequences of *trpL* mRNA and its corresponding amino acid sequence is shown on the left. Black arrowhead marks translation arrest at the start codon, while colored arrowheads point to the drug-induced arrest sites within the coding sequences of mRNAs used. Note that due to the large size of the ribosome, the reverse transcriptase used in the toeprinting assay stops 16 nucleotides downstream of the codon located in the P-site.

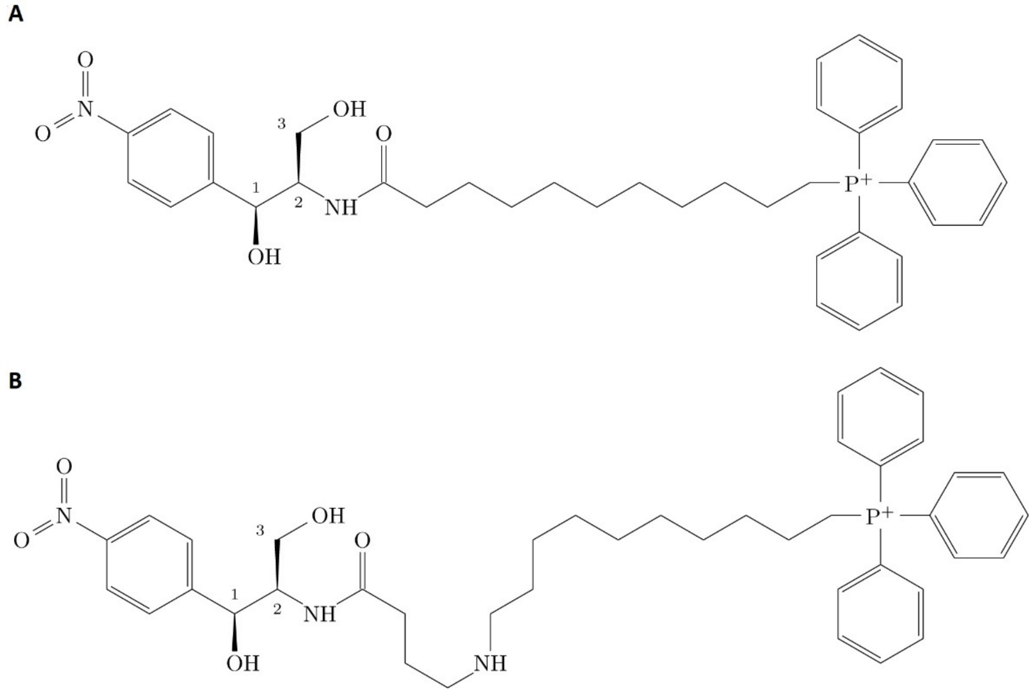

Figure S2. Triphenylphosphonium derivatives of chloramphenicol amine: CAM-C10-TPP (**A**) and CAM-C14-TPP (**B**). Atom numbering is based on [79].

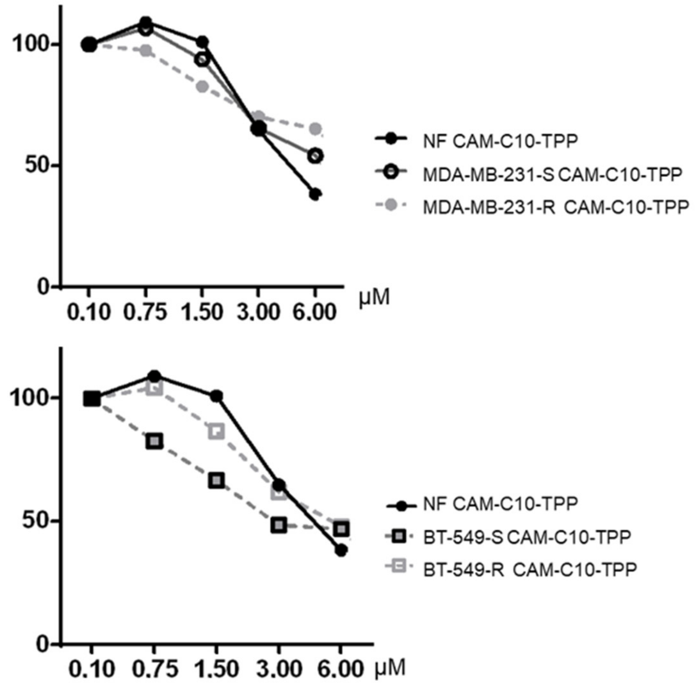

Figure S3. Effect of CAM-C10-TPP derivative on cell viability in normal (NF) and TNBC cells. Normal human fibroblasts (NF), MDA-MB-231 and BT-549 TNBC sensitive (S) or chemoresistant (R) cells were grown at 60% confluence for 3 days with the indicated concentrations of CAM-C10-TPP derivatives. Representative graphs show cell viability assays. Results are the mean of 3 independent experiments ($p < 0.05$).
